# Prevalence of undiagnosed dyslexia in African-American primary school children

**DOI:** 10.1038/s41539-023-00204-8

**Published:** 2023-12-02

**Authors:** Laura Cassidy, Kayla Reggio, Bennett A. Shaywitz, Sally E. Shaywitz

**Affiliations:** 1 Dyslexia Resource Center, Louisiana Key Academy, Baton Rouge, LA USA; 2 Yale Center for Dyslexia & Creativity, Department of Pediatrics, Yale University School of Medicine, New Haven, CT USA

**Keywords:** Education, Human behaviour

## Abstract

Dyslexia is among the most common neurodevelopmental disorders in children, yet despite its high prevalence all too frequently goes undiagnosed. Consequently dyslexic children all too often fail to receive effective reading interventions. Here we report our findings from a study using a teacher completed evidence-based dyslexia screener to first screen then test predominantly African-American children in grades kindergarten through second grade in two inner city public charter schools in New Orleans. Almost half (49.2%) of the children screened as at risk for dyslexia and of these the majority were found to be dyslexic on more detailed testing. Our results suggest that large numbers of African-American students with dyslexia may be overlooked in schools.

Dyslexia is the most common neurodevelopmental disorder in children-epidemiological studies indicate that dyslexia is highly prevalent, affecting one in five^1,2^, with boys and girls equally impacted^3^. Since its first description over a century ago^4^ and now in recent United States federal legislation, dyslexia is defined as “an unexpected difficulty in reading for an individual who has the intelligence to be a much better reader, most commonly caused by a difficulty in the phonological processing (the appreciation of the individual sounds of spoken language), which affects the ability of an individual to speak, read, and spell.”^5^. The unexpected nature of dyslexia is supported by empirical data which indicate that in typical readers IQ and reading are linked while in dyslexic readers IQ and reading diverge^2,6^. Functional brain imaging studies from laboratories around the world demonstrate a neural signature for dyslexia: an inefficient functioning of those neural systems responsible for automatic fluent reading located in posterior regions of the left hemisphere^7,8^.

Perhaps most importantly, we now know that the achievement gap between typical and dyslexic readers is evident as early as first grade and persists^1^. This recognition led to the development of an evidence based, inexpensive, and highly accurate dyslexia screener^9^ applicable for universal screening and in use in 49 states in the US, offering the possibility of identification of children at risk for dyslexia.

It has been almost 60 years since the publication of the Coleman report^10^, itself a part of the Civil Rights Act of 1964, documenting the increased prevalence of reading difficulties in African-American children. Since that initial report an extensive literature has confirmed the black–white achievement gap in reading. For example, data from the Early Childhood Longitudinal Study indicate that by the end of third grade there is a large black–white achievement gap in reading^11^. More recent studies confirm the black–white achievement gap in reading^12^. Interestingly, the term dyslexia does not appear in any of these studies focusing on the black–white achievement gap in reading, including data from NAEP noted below, which provides the best previous evidence for the high prevalence of dyslexia in African-American children. In fact, though first described over a century ago, dyslexia has not been used in discussions of the black–white achievement gap in reading.

The National Assessment of Educational Progress (NAEP) is often referred to as the “Nation’s Report Card.” Every two years NAEP reports reading and math scores of several thousand fourth and eighth graders from schools across the US. For more than a decade NAEP data have consistently demonstrated that more than half of black fourth graders perform below the NAEP Basic level in reading compared to about 20% of white students^13^. To a large extent this has been ignored, perhaps because the reading comprehension metric used by NAEP (most commonly presented as the percentages of students attaining NAEP reading achievement levels scored as Basic, Proficient and Advanced, above Proficient) has been difficult to relate to more readily understood reading measures. In an effort to better characterize NAEP achievement levels White and his associates^14^ examined a nationally representative sample of more than 1800 4th-grade students from 180 public schools with measures of decoding, sight word reading, and oral reading fluency. The oral reading fluency test measured accuracy, rate, and expression which are all components of reading fluency. Their data indicated that “significant percentages of the students who perform below the NAEP Basic level on the 4^th^ grade NAEP reading assessment have poor oral reading fluency and foundational skills.” It has not escaped our notice that the constellation of poor oral reading fluency and a weakness in decoding found in the children scoring below basic on the NAEP is also characteristic of dyslexia. Thus, for well over a decade. successive waves of NAEP reading data suggest that the large numbers of African-American students identified as reading below basic are who many knowledgeable educators would identify as having dyslexia.

## Screening results

Here we report the results of a study that used this teacher completed dyslexia screener to first screen children in grades kindergarten through second grade in two inner city schools. Children were screened for dyslexia using an evidence-based screener^15^ which follows the criteria for dyslexia screening in federal law^5^. The screener is designed as a series of questions posed to the teachers based on age appropriate skills dealing with the interface of oral and written language (phonological processing) and is not a test or assessment tool requiring a response by the student. Psychometrics are excellent with ROC Area Under the Curve (AUC) for kindergarten, first grade and second grade 0.81, 0.89, and 0.92 respectively^9^. Those screening at risk for dyslexia were then evaluated using measures consistent with the 2018 law defining dyslexia^5^ including detailed reading and intelligence tests. As shown in Tables 1 and 2, 246 children were screened for dyslexia, and 121 (49.2%) screened as at risk for dyslexia (Table 1). Of the children screened, 221, 90% were African-American, 8% Hispanic, and 2% white and 93% of the children screened were considered disadvantaged.

**Table 1:** Screening results.

Categories	Total (*n* = 246) (%)	African-American	Hispanic	White
At-risk	121 (49)	104	13	4
Not at risk	125 (51)	117	6	2

**Table 2:** Testing Results.

Categories	Total (*n* = 92) (%)	African-American (*n* = 81)	Hispanic (*n* = 7)	White (*n* = 4)
Consistent with dyslexia	57 (62)	53	1	3
Not dyslexic	7 (8)	7	0	0
English language learner	7 (8)	1	6	0
Indeterminate	21 (23)	20	0	1

## Testing results

Of the 121 children screened as at risk, we were able to obtain parental consent to test 92 (76.0%) and results are shown in Table 2. Of these 92 students, 57 (62%) had a profile consistent with dyslexia, and 53 of the 57 (93%) were African-American; 7 (8%) students did not have dyslexia, 7 (8%) students spoke Spanish as their primary language and 21 (23%) students were classified in the indeterminate category.

## Discussion

Explanations for this increased prevalence of reading disability in African-American children remain controversial and we will just touch on this topic briefly. For example, the Coleman et al original report concluded that a student’s family background influences his or her reading more than the school that student attends. In contrast, reanalysis of the original Coleman et al. data, using 21st-century statistical methods and modern computer resources points to 40% of the differences in reading achievement being explained by school characteristics rather than family influences^16^. More recent studies suggest that socioeconomic status represents a key determinant of black–white test score gaps^12^. Other studies suggest that imposing accountability on schools, for example in the No Child Left Behind federal legislation, results in improved reading scores for black children^17^. Ironically, current U.S. federal law may have unwittingly exacerbated the black–white achievement gap. Thus federal education law excludes from the definition of disability (and with this, exclusion from identification and intervention) disadvantaged children, many of whom are African-American. In the most recent iteration of IDEA^18^, within the definition of specific learning disability (SLD), the current term incorporating dyslexia, excludes children from a diagnosis of dyslexia who have “…environmental, cultural, or economic disadvantage (emphasis added).” Unfortunately this has harmed children living in poverty, many of whom are African-American. This has had the pernicious effect of encouraging educators to attribute poor reading to poverty rather than to screen for, identify and provide interventions for children with dyslexia.

Our current findings using well-established metrics, along with data from NAEP studies over the last decade, suggest that large numbers of African-American students may have dyslexia and are currently not diagnosed and are being overlooked in school. At the same time our results suggest a path forward to help these large numbers of boys and girls whom NAEP data identify as reading below basic and whom our data suggest are in fact children with dyslexia. Children with dyslexia who are struggling in school to read what their classmates seem to be reading so effortlessly, but who remain undiagnosed come to view themselves as “dumb.” These children would rather misbehave and be sent to the principal’s office than have to stand up and read aloud in front of their class. Once a diagnosis of dyslexia is made and shared with the child, teachers, and parents often observe a remarkable change—the child feels empowered, he knows his condition has a name, dyslexia, and this diagnosis means that while he may read slowly, he is bright and has no holes or lesions in his brain. This scenario contrasts with one where far too often the failure to diagnose dyslexia results in what is often referred to as the school to prison pipeline where about half the prisoners test as dyslexic, bright but struggling to read but have never been identified when they were in school and have no idea they may be dyslexic^19^.

We are in a positive new era where educators know not only how to effectively and efficiently screen for dyslexia using evidence-based screeners but in addition how to provide evidence-based interventions to treat children with dyslexia, interventions which might include placement in specialized schools such as the Louisiana Key Academy^6^ (pp. 347–358). The science of dyslexia has moved forward and now mandates that all children, including the large numbers of African-American children who may be dyslexic, receive the benefits of these advances in screening, diagnosis, and intervention.

These data have important implications for educators and policy makers. Regardless of the underlying explanation for the high prevalence of reading difficulties in African-American children, the fact remains that these children must be screened for dyslexia early and provided with effective, evidence-based interventions. Based on these data most of the large number of African-American children with reading difficulties may, in fact, have dyslexia, and the good news is that half a century of research has shown how best to provide effective interventions for dyslexic children.

## Methods

### Participants and screening

Children were recruited from kindergarten, first and second grade in two inner city public charter schools in New Orleans, Louisiana. More than 90% of the children were eligible for free lunch and were considered low income; 90% were African-American, 8% Hispanic and 2% white (Table 1). The study protocol was approved within the agreement between the New Orleans College Prep Organization and the Dyslexia Resource Center and conducted in accordance with the principles of the Declaration of Helsinki. All parents or legal guardian(s) of the children in the study provided written informed consent (and children provided assent where applicable) after any psychometric testing and possible side effects were fully explained. Consent/assent included permission for both research and publication in medical journals.

### Measures

In early spring 2018 children were screened for dyslexia using the evidence-based Shaywitz DyslexiaScreen^9^ which follows the criteria for dyslexia screening in federal law noted above. The screening was done in early spring of the school year by the child’s teacher. The teachers had an intensive training session on dyslexia by an experienced Certified Academic Language Therapist from the Dyslexia Resource Center. This screener is brief, completed independently by the teacher on a tablet and is based on that teacher’s observations and knowledge of the student, ideally after spending months with each child in the classroom.

If a student was identified as “at risk for dyslexia” consent was obtained from the school and the student’s parent before detailed individual testing was administered. The testing included the Kaufman Brief Intelligence Test (KBIT-2) which examines verbal and nonverbal intelligence^20^; the Test of Word Reading Efficiency (TOWRE-2)^21^ which evaluates single real word and pseudoword reading fluency; and the Comprehensive Test of Phonological Processing 2^nd^ edition (CTOPP-2)^22^ which examines the ability to blend and isolate parts of words. Oral reading fluency of connected text was assessed using the Dynamic Indicators of Basic Early Literacy Skills Oral Reading Fluency Test^23^ (DIBELS 8).

Briefly, the following general guidelines were used to categorize participants: dyslexic if their KBIT-2 IQ was 70 or above and all their reading-related measures (CTOPP-2, TOWRE-2, ORF) were unexpectedly low (either compared to the child’s ability or compared to the child’s age and grade. Low reading scores compared to ability are reading standard scores 15–20 points below the highest IQ, either verbal or performance; low reading scores compared to age are reading scores below a standard score of 90. Children were classified as not dyslexic if their KBIT-2 IQ score was 70 or above and any of their reading-related measures were in the average range. With these as general guidelines, the authors, all of whom have extensive experience evaluating and diagnosing dyslexia, reviewed each child’s data and by consensus^24^ classified the participants as (1) dyslexic, (2) not dyslexic, (3) English language learner (if the child’s primary language was not English), and (4) indeterminate, primarily those kindergarten children who had a very limited amount of testing.

### Reporting summary

Further information on research design is available in the Nature Research Reporting Summary linked to this article.

### Supplementary information

Reporting Summary

## Data Availability

The datasets generated during and/or analyzed during the current study are not publicly available due to containing information that could compromise research participant privacy/consent. We will share anonymized data with requesting authors for meta-analyses and other reviews; we will not require a Data Use Agreement.
